# Fabrication of date palm kernel biochar-sulfur (DPKB-S) for super adsorption of methylene blue dye from water

**DOI:** 10.1038/s41598-024-56939-w

**Published:** 2024-03-21

**Authors:** Marwa R. Elkatory, Murat Yılmaz, Mohamed A. Hassaan, Ahmed El Nemr

**Affiliations:** 1 Advanced Technology and New Materials Research Institute, SRTA-City, 21934, New Borg El-Arab City, Alexandria Egypt; 2 Department of Chemistry and Chemical Processing Technologies, Bahçe Vocational School, Osmaniye Korkut Ata University, 80000 Osmaniye, Turkey; 3 National Institute of Oceanography and Fisheries (NIOF), Kayet Bey, Elanfoushy, Alexandria Egypt

**Keywords:** Biochar, Date palm kernel, Methylene blue, Adsorption, Sulfur activation, Pollution remediation, Chemical engineering

## Abstract

A novel form of biochar was created by dehydration of Date palm kernel with 85% sulfuric acid. It was examined how the newly produced biochar (DPKB-S) affected the aqueous solution's capacity to extract Methylene Blue (MB) dye. The prepared DPKB-S was categorized by BET, BJH, FT-IR, SEM, EDX, DSC, and TGA analyses. The ideal pH for the MB dye adsorption by DPKB-S is 8. With 0.75 g L^−1^ of DPKB-S and an initial concentration of 50 ppm MB dye, Date Palm Kernel Biochar-Sulfur (DPKB-S) had the highest removal percentage of 100%. The Langmuir and Freundlich isotherm models were used to investigate the collected data. Freundlich model is the model that best covers MB dye adsorption in DPKB-S at low concentrations (0.75–1.25 g L^−1^) and the Langmuir model at high concentrations (1.5–1.75 g L^−1^). The Langmuir model maximum adsorption capacity (*Q*_*m*_) of the DPKB-S was 1512.30 mg g^−1^. Furthermore, a variety of error function models were applied to investigate the isotherm models derived data, including Marquardt’s percent standard deviation (MPSD), the sum of absolute errors (EABS), the sum of the errors squared (ERRSQ), root mean square errors (RMS), Chi-square error (X^2^), the average relative error (ARE), average percent errors (APE), and hybrid error function (HYBRID). Kinetic data were calculated by intraparticle diffusion (IPD), pseudo-second-order (PSO), pseudo-first-order (PFO), and film diffusion (FD) models. A PSO rate model with a strong correlation (*R*^2^ = 1.00) largely regulated the adsorption rate. The removal mechanism of MB dye by DPKB-S is based on the principle that these positively charged dyes are attracted by electrostatic attraction forces due to the growth in the number of negatively charged regions at basic pH value. According to the results, DPKB-S shows promise as an affordable and competent adsorbent for the adsorption of MB dye. It can be used frequently without experiencing a discernible decrease in adsorption efficiency.

## Introduction

Due to ongoing pollution of the waters already present in various parts of the world, there is growing anxiety about water shortage in some parts of the world. Dyes^1,2^, heavy metals^3–5^, medical drugs^6,7^, and Crude oil^8^ are examples of chemical substances that seriously damage the environment. Wastewater from homes, businesses, and hospitals releases these chemicals into the environment.

Since dyes are coloured, it is particularly easy to find them in wastewater. Synthetic dyes are the most popular kind of dyes used in leather, textiles, paint, and other industries^9^. Since most colours are non-biodegradable, carcinogenic, and toxic, these stresses harm both human health and the ecological balance^10,11^. An average of 0.7–2.0 × 10^5^ tons of crude dyestuffs are discharged into water bodies annually, accounting for approximately 10–20% of the total^12^. When it comes to synthetic dyes, azo dyes are the most versatile, and largest, and come in the greatest number of colours. When these substances are overused, carcinogenic compounds are produced^13^.

A variety of techniques are available for treating dyehouse effluent, but the most common ones are as follows: chemical oxidation^14^, biological treatment^15^, coagulation/flocculation^16^, photo-degradation^17^, electrochemical treatment^18^, and adsorption treatment^19,20^.

One of the most popular methods for getting rid of dyes is the adsorption method utilising activated carbon (AC) because of its high effectiveness. However, developing more economical and more effective adsorbents instead of commercial activated carbons, which are expensive both in production and processing, is attracting more and more attention from scientists every day^21,22^. Production of biochar is becoming more and more popular as a less expensive and greener substitute. In addition, biochar keeps limited resources from being squandered since it is produced using biomass and residues as raw materials. The term “biochar” describes carbonaceous solids obtained through gasification or biomass pyrolysis under nitrogen at temperatures higher than 350 °C^22^. In their investigation, Güzel et al.^23^ discovered that the production of commercial activated carbon is typically more expensive than the production of biochar. Biochars are inexpensive, but they also have advantages like producing high-value adsorbents, lowering secondary environmental pollution, and being renewable^24^. Furthermore, the amount of CO_2_ released into the atmosphere is reduced when biochar is used as an adsorbent^25^. Though biochars have more functional groups on their carbonaceous surface than ACs, they have lower surface areas and pore volumes^26^. To improve the practical uses of biochars for removing colourants from wastewater, it is feasible to chemically modify the surfaces of the biochars to increase the number of functional groups. The overall objective of biochar modifications such as oxidation, mineral impregnation, surface reduction, and nanoscale creation is to increase biochar’s adsorption capability^27^. To improve the biochar’s capacity, amino groups are introduced to the adsorbent's pores during the mineral element impregnation process^28^. Using a variety of oxidising chemicals (NaClO, H_2_O_2_, NH_3_·H_2_O, KMnO_4_ or (NH_4_)_2_ S_2_O_8_), bases (NaOH or KOH), and acids (H_2_SO_4_, H_3_PO_4_ or HNO_3_), the surface oxidation approach aims to increase the functional groups' number^29–31^.

Numerous research have been conducted on the use of ACs derived from biomass to remove different types of pollutants. Orange peel^32^, Coconut Shell^33^, potato^34^, tea waste^35^, rice straw^36^, mandarin peel^37^, watermelon peel^38^, olive stone^39^, peanut husk^40^, bamboo^41^, and wheat straw^42^ are some of these biomasses.

Biomass obtained from date palm trees is a good option to use in the production of effective AC due to their advantages such as being abundant in Mediterranean countries, being cheap, being easily biodegradable, having low toxicity and having a strong affinity towards pollutants. Date palm material typically contains 15–35% lignin, 20–35% hemicellulose, and 40–50% cellulose in that order. The average percent elemental compositions of hemicellulose and cellulose are 49.4 wt% oxygen, 44.4 wt% carbon, and 6.2 wt% hydrogen^43^. Lignin is a three-dimensional polymer consisting of phenyl propane units connected by C–O–C or C–C linkages. As a result, its elemental composition has a lower percentage of oxygen (32 wt%) and a higher percentage of carbon (62 wt%)^44^.

No study has been found in the literature on the Methylene Blue (MB) dye adsorption from water by biochar obtained from date palm kernel materials by dehydration with concentrated H_2_SO_4_.

This study assessed the effectiveness of date palm kernel biochar-sulfur (DPKB-S), a cheap agricultural waste material made from date palm kernel, in eliminating Methylene Blue dye from an aqueous environment. DPKB-S was dehydrated by boiling the kernels in 50% H_2_SO_4_. As the conditions for removing MB dye from wastewater, variables like the adsorbate’s initial concentration, the solution pH, solution temperature, and the impact of the adsorbent dose were investigated. The maximum adsorption capacity and the structure of the adsorption were ascertained by analyzing the isotherms, thermodynamics and kinetics for the elimination of MB dye by DPKB-S adsorbents.

## Materials and methods

### Instrument and materials

Date palm kernel was purchased from a local market in Alexandrian and utilised as the raw material to create Date Palm Kernel Biochar-Sulfur (DPKB-S), an adsorbent substance. The supplier of sulfuric acid (H_2_SO_4_, Purity 98%) was the Sigma Aldrich business. The MB dye (Aldrich, USA) concentration was measured using an analytical Jena digital spectrophotometer (SPEKOL1300 UV/Visible spectrophotometer) in conjunction with 1 cm optical path glass cells, a shaker (JSOS-500) for mixing procedures, and a pH metre (JENCO 6173) for pH surveys. The adsorption–desorption isotherm of DPKB-S was measured in the N_2_ environment. Using an instrument (BELSORP—Mini II, BEL Japan, Inc.), the surface area, pore size and pore distribution of DPKB-S were determined^45,46^. Monolayer volume (*V*_*m*_) (cm^3^) (STP), surface area (S_BET_) (m^2^/g), average pore diameter (MPD) (nm), total pore volume (*p*_*0*_/*p*_*0*_) (cm^3^/g) and energy constant (*C*) values of DPKB-S were obtained by modelling of the adsorption–desorption graph. The microporous surface area (*S*_*mi*_), mesoporous surface area (*S*_*mes*_), mesoporous volume (*V*_*mes*_), and microporous volume (*V*_*mi*_) of DPKB-S were calculated by the Barrett–Joyner–Halenda (BJH) model. The calculations were carried out with the software of the BELSORP analysis programme. Using the BJH approach, the pore size dispersion was also ascertained from the desorption isotherm^47^. An investigation of the form of the biochar surface was conducted using a scanning electron microscope (SEM; QUALITY 250). Fourier Transform Infrared (FTIR) spectroscopy (VERTEX70) and the ATR unit model V-100 were used to investigate the functional groups on the surface of DPKB-S. IR-observable functional groups on the DPKB-S surface were identified in the 400–4000 cm^−1^ wavenumber region using FTIR spectroscopy in combination with the platinum ATR unit. Employing the SDT650-Simultaneous Thermal Analyzer apparatus, thermal analyses were conducted at a ramping temperature of 10 °C/min throughout a temperature range of 50–1000 °C.

### DPKB-S preparation

Date palm kernels, which were purchased from a nearby market and composted, were utilised as the carbonaceous starting material in the biochar synthesis. The Date Palm Kernels were extensively cleansed with tap water many times to remove any dust, and they were thereafter dried in a furnace at 120 °C for twenty-four hours before being ground and pulverised. A total of 100 g of powdered Date Palm Kernel was heated at 250 °C in 500 mL of 85% H_2_SO_4_ solution for 5 h, then diluted with distilled water, filtered and then washed with distilled water until pH 7. The DPKB-S was then cleaned with EtOH and dried at 120 °C in a furnace. Biochar with the designation DPKB-S was produced as a consequence of this reaction.

### Batch adsorption experiment

A batch adsorption experiment was used to assess the sorption capacity, thermodynamic, and kinetic properties of DPKB-S. A reasonable quantity of flasks with 100 mL of MB dye solutions at various starting concentrations and DPKB-S at various weights were shaken for a predetermined amount of time at 200 rpm. Solution pHs were raised or lowered to the appropriate levels with 0.1 M NaOH or HCl. Furthermore, during the adsorption equilibrium investigations, the pH of the solution was maintained at the intended level. Taking a sample (0.1 mL) from the solution at regular intervals (removed from the adsorbent) allowed for the determination of the MB dye concentration using a spectrophotometer set at λ_max_ = 665 nm. The *q*_t_ of DPKB-S was calculated using Eq. (1).

1 $${q}_{t}=\frac{\left({C}_{0} -{ C}_{t}\right)}{W} V$$

where *C*_*0*_ (mg/L) is the MB dye initial concentration; *C*_*t*_ (mg/L) is the remaining MB dye concentration at the end of time *t*; *q*_*t*_ (mg/g) is the adsorption capacity of DPKB-S at time t; *W* (g) is the mass of the DPKB-S and *V* (L) is the volume of the MB dye solution.

To examine the impact of pH on the adsorption of MB dye ions by DPKB-S, studies were achieved at different pH values (2.0 to 10) by adding 0.1 g DPKB-S to 100 mL of solutions containing 100 ppm MB dye. The mixtures were agitated for 150 min at 200 rpm when the mixtures were at room temperature.

MB dye solutions with varying initial concentrations (50–150 ppm) were made, and isotherm measurements and the effect of DPKB-S dose on the adsorption of MB dye ions were investigated. Intervals between 0.75 and 1.75 g/L of DPKB-S doses and MB dye solutions with diverse starting concentrations were used to measure the MB dye concentrations. The mixtures were agitated at 200 rpm and 25 °C. Every adsorption investigation was carried out in triplicate, and the results are presented as an average.

In the course of adsorption thermodynamic measurements, 100 mL Erlenmeyer conical flasks holding 100 mL of MB dye concentration (100 ppm) were filled with 0.1 g of DPKB-S (1.0 g/L). In an orbital shaker, the mixture was agitated at 200 rpm, under ideal circumstances (pH of 8.0, contact time of 150 min), and at preset temperatures (25–45 °C). The DPKB-S was separated by centrifugation, and the UV–vis spectrophotometer was used to measure the amount of leftover MB dye in the supernatant. To ensure system reproducibility, every adsorption thermodynamic experiment was carried out three times, and the mean results were utilised in the computations.

### Theoretical background

According to the Langmuir isotherm model^48^, there is no adsorbate movement along the surface’s plane and adsorption takes place on a surface in a monolayer^49^. The Langmuir equation^50–52^ is expressed in linear form in Eq. (2) (Table 1). The expressions qe (mg/g), *C*_e_ (mg/L), *Q*_m_ (mg/g), and *K*_a_ (L/mg) represent the equilibrium adsorption sorption energy, equilibrium concentration, and maximum adsorption capacity of the monolayer, respectively. Equation (3) illustrates the linear version of the Freundlich model^53^. The adsorption density, or surface heterogeneity, is represented by 1/*n*, which gets increasingly heterogeneous as it gets closer to zero. The relative adsorption capacity is stated in *K*_F_ (Table 1).

**Table 1:** The models for error analysis, kinetic and isotherm models^52^.

Model name	Equation	Equation
Langmuir	$\frac{{C}_{e}}{{q}_{e}}=\frac{1}{{K}_{a}{Q}_{m}}+\frac{1}{{Q}_{m}}{C}_{e}$	(2)
Freundlich	$log{q}_{e}=log{K}_{F}+\frac{1}{n}log{C}_{e}$	(3)
Pseudo-first-order	$log\left({q}_{e}-{q}_{t}\right)=log\left({q}_{e}\right)-\frac{{k}_{1}}{2.303}t$	(4)
Pseudo-second-order	$\left(\frac{t}{{q}_{t}}\right)=\frac{1}{{k}_{2}{q}_{e}^{2}}+\frac{1}{{q}_{e}}t$	(5)
Intra-particular diffusion	${q}_{t}={K}_{dif}{t}^{1/2}+C$	(6)
Film	$ln \left(1-F\right)= -{k}_{FD}\times t$	(7)
APE	$APE\left(\%\right)=\frac{100}{N} \sum_{i=1}^{N}{\left|\frac{{q}_{e,isotherm}-{q}_{e,calc}}{{q}_{e,isotherm}}\right|}_{i}$	(8)
HYBRID	$HYBRID=\frac{100}{N-P} \sum_{i=1}^{N}{\left|\frac{{q}_{e,isotherm}-{q}_{e,calc}}{{q}_{e,isotherm}}\right|}_{i}$	(9)
Chi-square	${X}^{2}=\sum_{i=1}^{N}\frac{{\left({q}_{e,isotherm}-{q}_{e,calc}\right)}^{2}}{{q}_{e,isotherm}}$	(10)
MPSD	$MPSD=100 \sqrt{\frac{1}{N-P} \sum_{i=1}^{N}(\frac{{q}_{e,calc}-{q}_{e,isotherm}}{{q}_{e,isotherm}}{)}_{i}^{2}}$	(11)
EABS	$EABS=\sum_{i=1}^{N}{\left|{q}_{e,calc}-{q}_{e,isotherm}\right|}_{i}$	(12)
RMS	$RMS=100 \sqrt{\frac{1}{N}\sum_{i=1}^{N}(1- \frac{{q}_{e,calc}}{{q}_{e,isotherm}}{)}^{2}}$	(13)

The Eq. (4)^54^ is a standard Lagergren first-order model equation, where the PFO adsorption rate constant, *k*_1_, (L/min), and the adsorption capacity at time *t* (min) are represented as qt (mg/g). The graph of log(*q*_e_ − *q*_t_) over time should provide a linear connection, from which *k*_1_ and the expected *q*_e_ may be obtained using the plot’s slope and intercept, respectively. Ho et al.^55^ described Eq. (5) as a linear PSO model. The PSO adsorption rate constant, *k*_2_ (g mg^−1^ min^−1^), expressed in this model reflects the PSO adsorption rate constant used to assess the initial sorption rate (*h*), which is equal to *k*_2_*q*_e_^2^. The values of *k*_2_ and *q*_e_ may be found using the intercept and slope of the *t*/*q*_t_ against t plots, respectively. Here, we explore the possibility of intra-particular diffusion using Eq. (6)^56,57^. The barrier to external mass transfer increases in tandem with the growth of the intercept *C* value. The intra-particle diffusion rate constant, expressed as *K*_dif_ (mg g^−1^ min^−1/2^), may be found simply by observing the slope of the regression line that plots *q*_t_ vs *t*^1/2^. Film diffusion (*FD*) is represented by Eq. (7), where *F* = *q*_t_/*q*_e_ (Table 1)^58^. Film diffusion rate coefficient (L/min) and fractional attainment of equilibrium are represented by F and kFD, respectively. Plotting ln (1-*F*) versus* t*, with a zero intercept, shows that the diffusion via the liquid layer surrounding the biosorbent is governed by the sorption process kinetics^52,59^.

The agreement between the experimental and predicted data derived from the isotherm model curves is stated by the Eq. (8), which stands for mean percent error (APE)^60^. For the hybrid fractional error function, see Eq. (9)^61,62^. The Chi-square error, or *X*^2^, is given by Eq. (10)^62^. Marquart's percentage standard deviation (MPSD) is expressed in Eq. (11)^60^. The total absolute error (EABS) is given by the following Eq. (12)^60^. The root mean square errors (RMS) may be computed using Eq. (13) (Table 1)^60^.

### Statement for the use of plants

In this study, Experimental research and field studies on plant material (Date palm kernel), including the collection of plant waste material, comply with relevant institutional, national, and international guidelines and legislation.

## Results and discussion

### DPKB-S characterization

**Figure 1:**
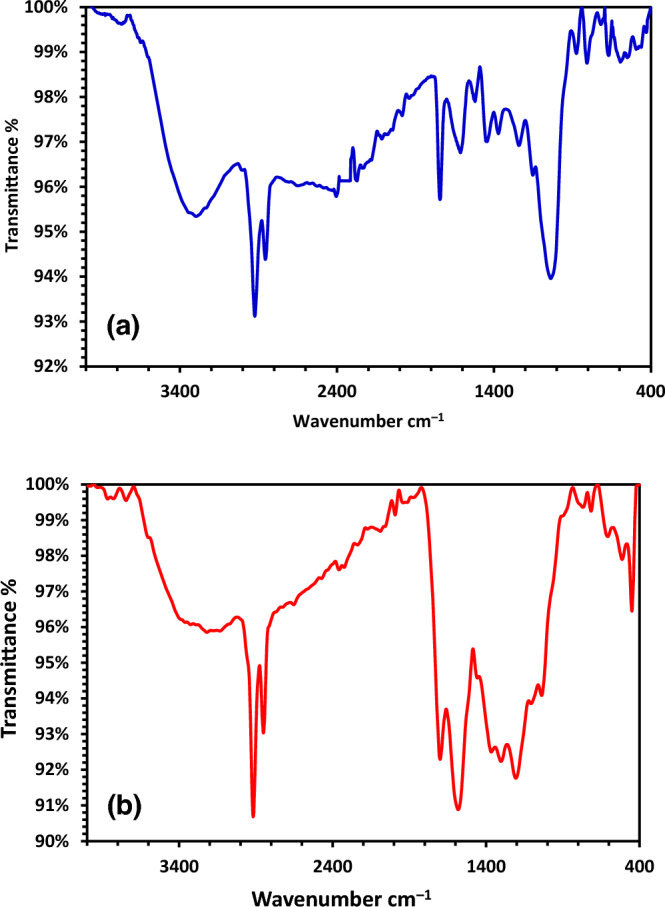
FTIR graphs of (**a**) Date palm kernel and (**b**) DPKB-S.

Using FT-IR spectroscopy, the functional groups present on the surface of the resulting DPKB-S adsorbent were identified. The FTIR graph of the raw Date palm kernel and the FTIR graph of the DPKB-S were compared, as shown in Fig. 1a,b. The FT-IR spectra of the materials show changes in their functional groups. The stretching oscillation of the O–H present in the Date palm kernel and DPKB-S is shown by the band between 3294.72 and 3135.08 cm^−1^ (Fig. 1). The presence of –CH_2_ stretching groups in date palm kernel is suggested by the high absorption peaks between 2923.29 and 2856.04 cm^−1^ (Fig. 1a). These groups were enlarged in DPKB-S and appeared at 2917.13 and 2850.95 cm^−1^ (Fig. 1b). The C=O stretching of the ester groups in the Date palm kernel is responsible for the high absorption band at 1742.87 cm^−1^ (Fig. 1a). This band was later transformed into a carboxyl group in DPKB-S at 1699.31 cm^−1^ (Fig. 1b). Nevertheless, the strength at 1699.31 cm^−1^ increased when DPKB-S was compared to raw Date palm kernel, indicating that sulphoric acid treatment may increase the carbonyl (C=O) group. The bands at 1613.23 cm^−1^ suggest that the *β*-ketone’s C=O stretching oscillation was nearly existent in the Date palm kernel. This oscillation shifted to 1581.85 cm^−1^ in DPKB-S with high intensity, and it might also be a stretching vibration of –C=C– in DPKB-S (Fig. 1b). The Date palm kernel’s C–O functional group is shown by the peaks at 1442.66 and 1371.75 cm^−1^. This group was replaced by the band at 1457.07–1205.40 cm^−1^ in DPKB-S, which displayed the sulfonyl group (S=O) stretching vibration (Fig. 1b). Additionally, the development of peaks at 1107.24 and 1037.97 cm^−1^ was facilitated by the dehydration process with H_2_SO_4_. These peaks resulted from the production of –SO_3_H and S=O groups in DPKB-S. These bands show that the Date palm kernel treatment with H_2_SO_4_ results in the creation of the DPKB-S. The Date palm kernel showed a more noticeable rise in the –C–O–C– asymmetric stretching functional group at 1038 cm^−1^ (Fig. 1a), compared to DPKB-S, which showed a partly weaker increase^63–66^.

**Figure 2:**
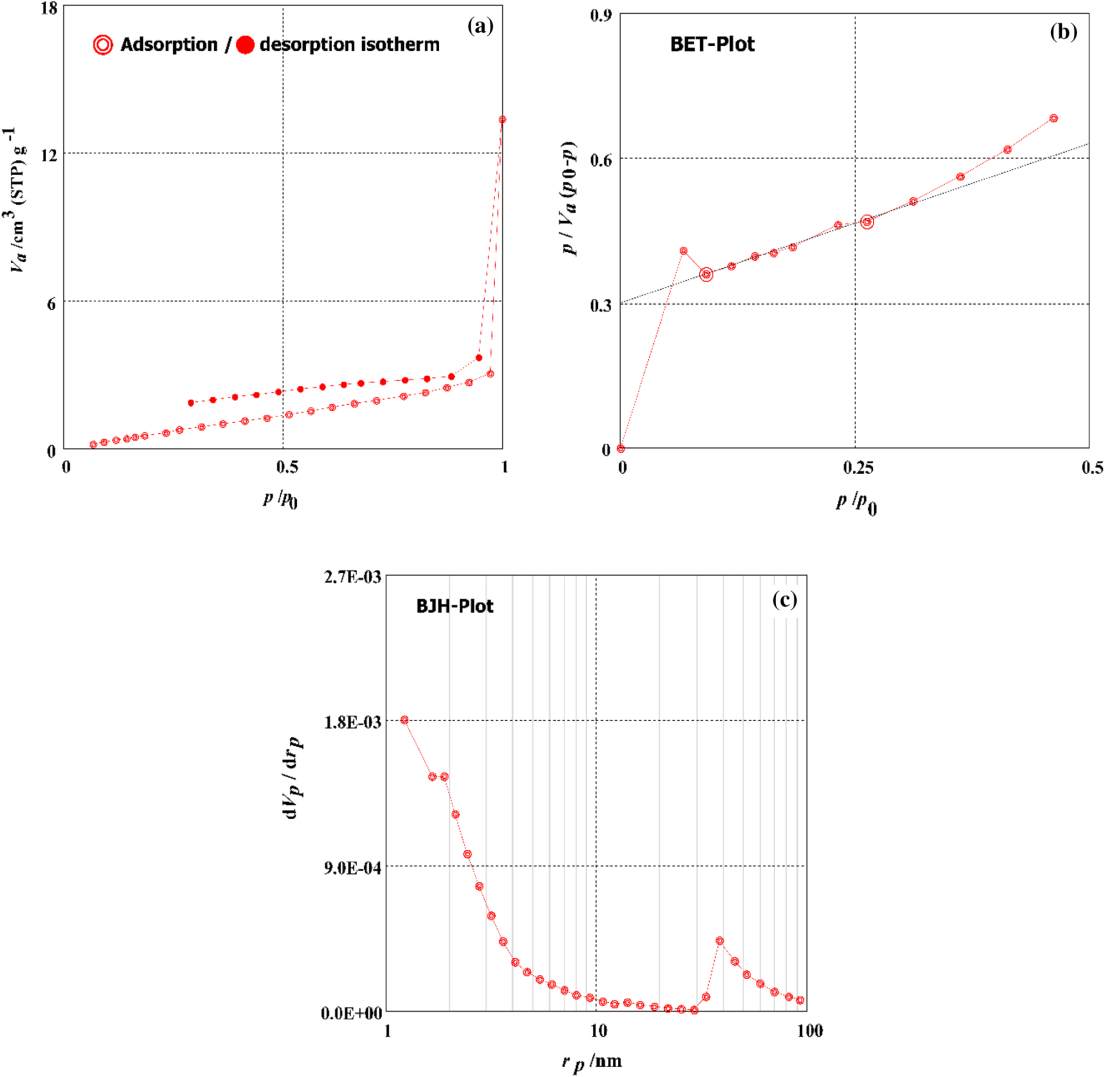
(**a**) Graph of N_2_ adsorption–desorption, (**b**) graph of the BET, (**c**) graph of the BJH of the DPKB-S.

To find out how H_2_SO_4_ affected the DPKB-S's surface characteristics, the N_2_ adsorption–desorption isotherm of the product was studied. The BET and BJH methods were used to compute the specific surface area and mesopore area, respectively. Figure 2 shows the textural properties of DPKB-S, including BET-specific surface area, mass of mesopores, mesopore area, total volume of pores, mesopore distribution peak, average pore diameter, and monolayer volume. The DPKB-S has a relatively tiny BET-specific surface area of 4.525 m^2^/g. DPKB-S had a monolayer volume value of 1.0395 cm^3^ (STP) g^−1^. DPKB-S has a total volume value of 1.5439 × 10^−2^ cm^3^/g. DPKB-S had mean pore diameters of 13.649 nm. The values of 4.623 m^2^/g, 1.6302 × 10^−2^ cm^3^/g, and 1.22 nm were found to be the mesopore volume, meso surface area, and mesopore distribution peak values of DPKB-S, respectively.

**Figure 3:**
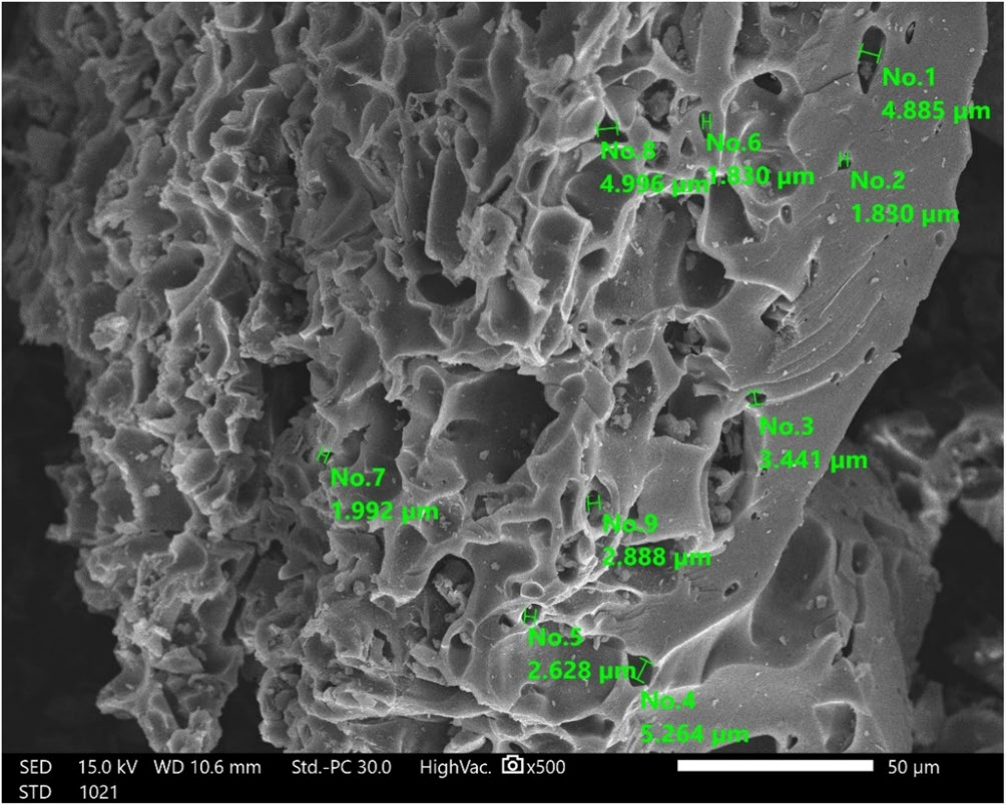
SEM image of DPKB-S using high vacuum SEM at magnification × 500 and 15.0 kV.

The DPKB-S is shown in SEM pictures in Fig. 3, where it is clear that it is clean and free of impurities. The DPKB-S pore structure remained unharmed by the intense sulfuric acid treatment. The average size of The DPKB-S is ranged between 1.830 to 5.246 μm (Fig. 3).

The DPKB-S adsorbent chemical composition was studied using scattered X-ray spectrometry (EDX). The percent of each element is presented in Table 2, which indicates that, in addition to carbon, which makes up 60.12% of the sample, there is around 1.10 and 374.2% of oxygen and sulphur, respectively.

**Table 2:** EDX results of prepared DPKB-S.

Elements	DPKB-S	
	Wt%	At%
C	60.12 ± 0.39	67.32 ± 0.43
O	37.42 ± 0.73	31.46 ± 0.62
S	1.10 ± 0.07	0.46 ± 0.03
Na	1.20 ± 0.10	0.70 ± 0.06
Ca	0.17 ± 0.04	0.06 ± 0.01
Total	100.00	100.00

**Figure 4:**
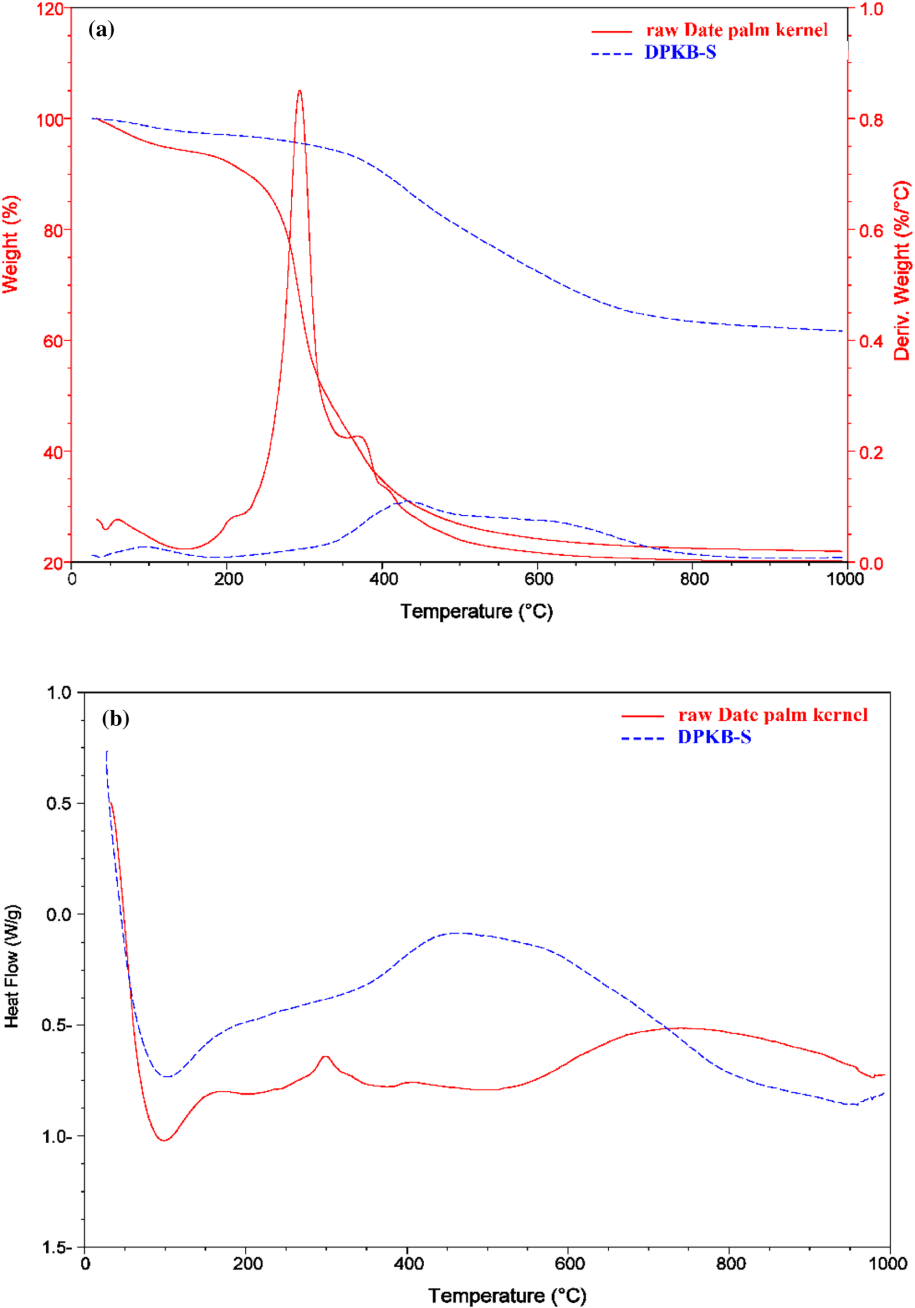
(**a**) Graphs of DTA and TGA, and (**b**) graph of DSC of the date palm kernel and DPKB-S.

The impact of structural variations on the operating temperature and degradation behaviour of the DPKB-S samples and raw date palm kernel was assessed using thermal gravimetric analysis (TGA). Every sample was cooked from 50 to 1000 °C in a N_2_ atmosphere. Figure 4 displays the TGA, Differential Thermal Analysis (DTA) and Differential Scanning Calorimetry (DSC) analytical curves for raw date palm kernel and DPKB-S. The first weight reduction was caused by the evaporation of water in the raw Date palm kernel and DPKB-S, and it peaked before 150 °C. Raw Date palm kernel and DPKB-S lost weight as a result of the breakdown of many acidic oxygen functional groups that occurred as the temperature rose beyond 150 °C. Moreover, acidic groups break down at different temperatures. For example, phenol breaks down at a greater temperature than lactones, anhydrides, and carboxylic groups. Raw Date palm kernel exhibits a high weight loss at temperatures up to 350 °C and the final weight loss occurred between 350 and 600 °C. DPKB-S shows two weight losses at temperatures between 150–500 and 500–900 °C, which explains the higher stability of DPKB-S compared to the raw Date palm kernel. TGA curve of DPKB-S converged at temperatures > 500 °C due to carbon breakdown in biomass. At the finishing temperature, various weight loss percentages of 75.79 and 37.65% were obtained for Raw Date palm kernel and DPKB-S, respectively, indicating the greater stability of DPKB-S.

DTA graph of DPKB-S and raw Date palm kernel is illustrated in Fig. 4a. The DTA curve of the raw Date palm kernel (red) peaked at three points at temperature (*T*_*f*_, 61.18, 294.09, and 377.94 °C), while the curve of DPKB-S (blue) peaked at two points at temperature (*T*_*f*_, 94.57 and 432.87 °C) (Fig. 4a). As can be seen from the DTA curve to produce DPKB-S adsorbents from raw Date palm kernel, dehydration of raw Date palm kernel (red) showed two well-resolved degradation bands. The degradation bands of raw Date palm kernel (red) decreased from three to two at higher temperatures after treatment with 85% H_2_SO_4_, demonstrating that the degree of degradation was strongly affected by H_2_SO_4_ treatment.

DSC may be used to compare materials based on thermal transitions. Figure 4b (blue) depicts the DSC graph of DPKB-S (red) and raw date palm kernel (red). The crystallisation temperatures (*T*_C_) of date palm kernels are 100 degrees Celsius, while DPKB-S displays *T*_C_ values ranging from 183.55 to 950.96 °C. When the temperature rises, DPKB-S melts at 521.72 °C, while date palm kernel melts at 152.59 and 292.53 °C. A lower *T*_m_ was shown by date palm kernel, whereas the highest *T*_m_ was shown by DPKB-S. The grains became more crystalline due to the higher transitional temperatures, which improved both their structural stability and resistance to gelatin disintegration.

#### XRD characterization of DPKB-S

**Figure 5:**
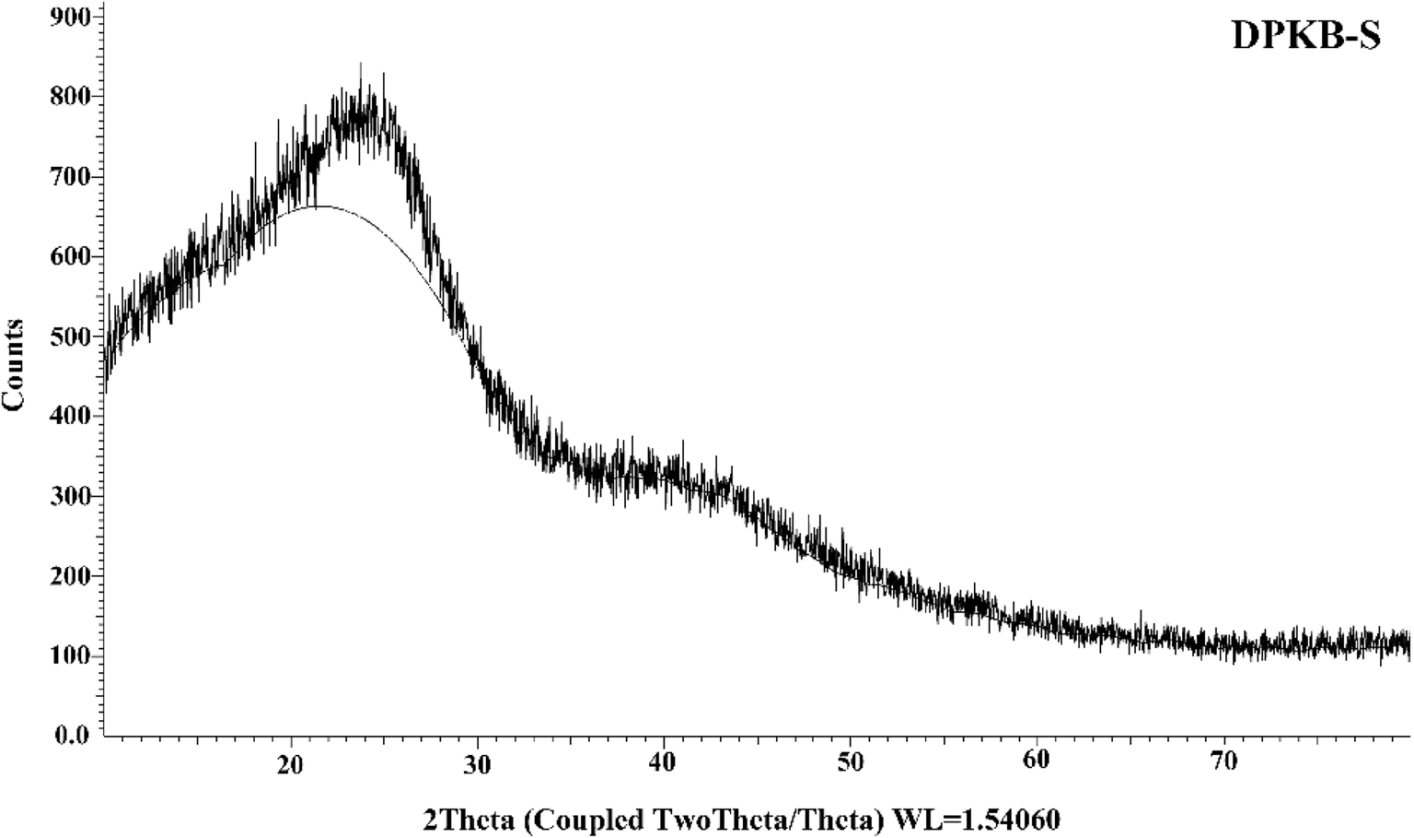
XRD graph of fabricated DPKB-S biochar.

The DPKB-S XRD is shown in Fig. 5 and shows an amorphous carbon structure with arbitrarily oriented aromatic sheets. A tiny peak is located around 2θ = 43.68, and a wide peak is indexed as the C (002) diffraction peak in the area of 2θ = 10–30. This might point to a variety of inorganic materials, primarily quartz and albite^67,68^.

### Adsorption of MB dye on date palm kernel biochar-sulfur (DPKB-S)

#### Impact of pH

**Figure 6:**
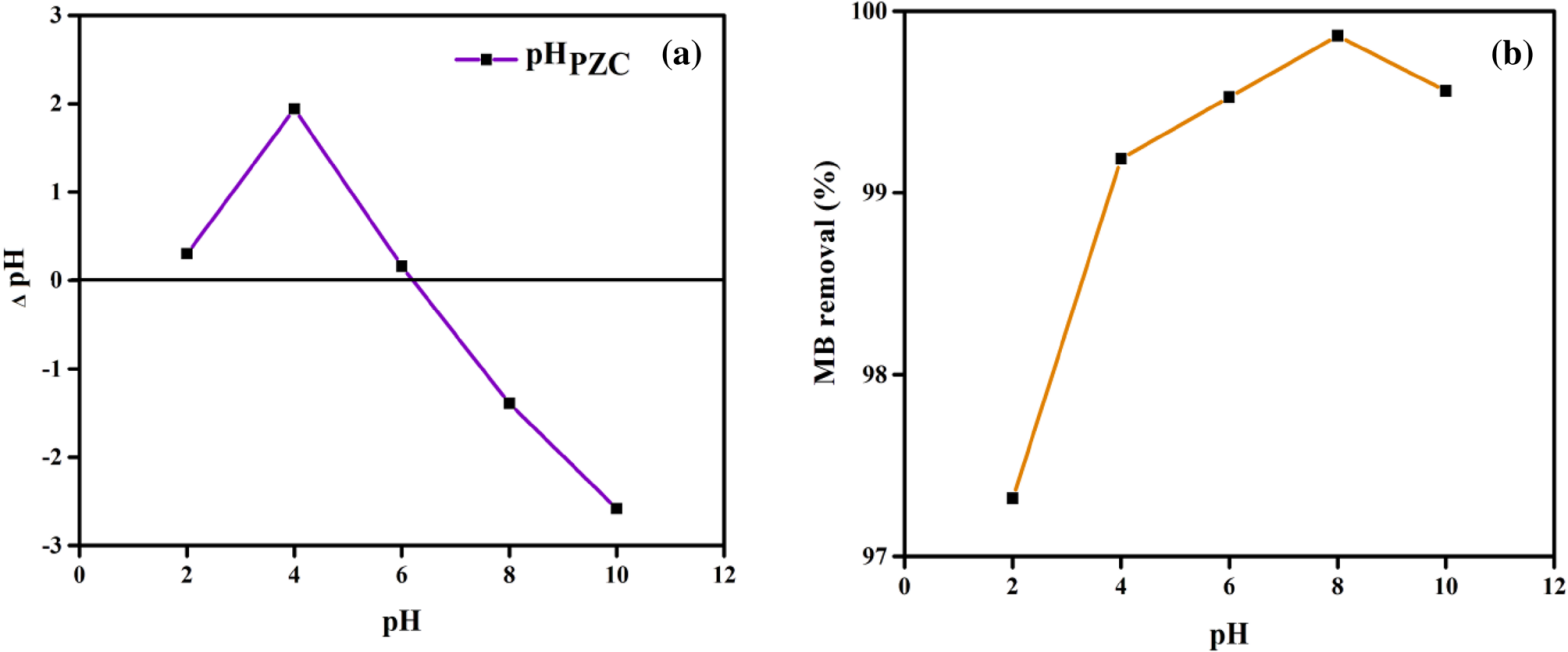
Sorption of MB dye onto DPKB-S as a function of (**a**) pH_ZPC_, (**b**) pH on the MB dye removal % (*C*_0_ = 100 ppm, DPKB-S = 1.0 g L^−1^, temp. = 25 °C).

The amino, hydroxyl, and carboxyl groups on the surface of DPKB-S biochar are impacted by the pH of the solution, which has an important effect on the adsorption process^20^. The adsorption mechanism of MB dye onto DPKB-S was studied by the varying pH of the MB dye solution from 2 to 10 at fixed initial MB dye concentration (100 ppm), contact time (180 min), adsorbent dose (1.0 g L^−1^), and temperature (25 °C). The graph shown in Fig. 6a indicates that the point of zero charge (pH_PZC_) is 6.2. When the solution pH was below pH_PZC_, the active sites on the biosorbent surface were positively charged, and when it was above it, they were negatively charged. Similar results were reported by El Nemr et al. in their study on the removal of acid yellow 11 dye^19^.

The pH study is displayed in Fig. 6b, which demonstrates that for MB dye adsorption using DPKB-S, pH 8 produced the maximum MB dye removal (99.9%). As seen in Fig. 6b, MB dye removal increased from 97.3 to 99.2% with increasing pH from 2 to 4. The increase in MB dye removal continued with increasing pH from 4 to 8, but then a decrease was observed with increasing pH from 8 to 10. Jabar et al.^69^ also examined the pH-dependent change in the removal of crystal violet dye, another azo dye, and observed a continuous increase in adsorption removal by increasing the pH value from 1 to 9. Then it shows a slight decrease^69^. Jabar et al.’s study on the adsorption of the Methylene Blue dye revealed that increasing the pH of the water from 2 to 8 enhanced the adsorption capacity^70^ from 50 to 250 mg g^−1^. The ideal pH for MB dye adsorption for DPKB-S was discovered to be 8. When MB dye molecules ionized in water, cations were created that competed with protons (H^+^) in the low pH aqueous solution for accessible anionic functional groups on the surface of DPKB-S. Protonation of the aqueous solution relaxed and the amount of MB dye adsorbed rose as the pH value rose from 2 to 8 (Fig. 6b). The increase in the percentage of MB dye adsorbed and MB dye uptake when the pH value rose from 2 to 8 might be attributed to an increase in the electrostatic attraction between the cationic dye and the anionic surface charge of the DPKB-S adsorbent^71^.

#### Contact time impact

**Figure 7:**
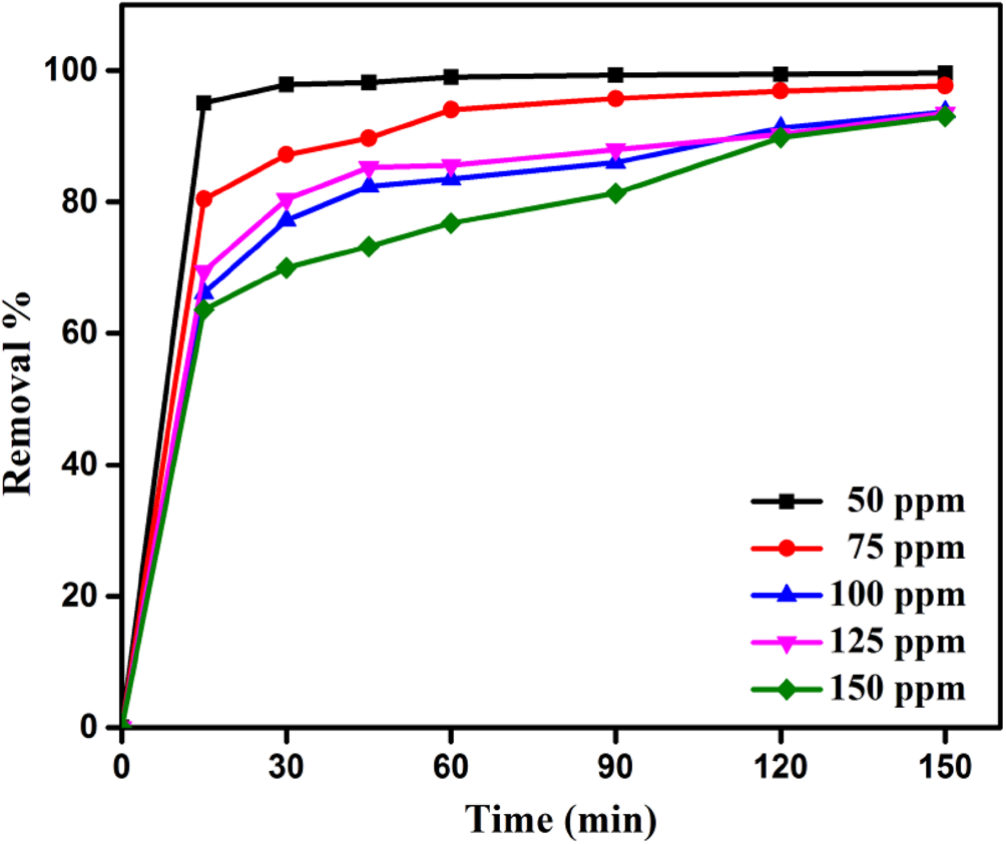
The MB dye removal % using DPKB-S as an adsorbent (MB dye = (50–150 ppm), DPKB-S dose = 0.75 g L^−1^, Temp. = 25 °C).

For the MB dye and DPKB-S adsorbent to interact as needed, contact time is crucial. DPKB-S at pH 8 was utilised, with the initial MB dye concentration varied from 50 to 150 ppm, to examine the impact of contact duration. Figure 7 shows that the adsorption process occurs rapidly in the first 15 min and proceeds steadily thereafter. 64–95% of the entire adsorption of the MB dye occurs during the first 15 min of adsorption (Fig. 7). The MB dye was continuously adsorbed as the duration of contact increased. Depending on the MB dye initial concentration (50, 75, 100, 125, and 150 ppm), after 150 min, the removal was 99.6, 97.7, 93.7, 93.5, and 93.0%, respectively.

The removal of MB dye from solutions with low initial concentration by DPKB-S will be high since the dye concentration will be low in empty active sites. However, when removing MB dye from DPKB-S adsorbent with a high initial concentration, the elimination percentage stays low because the active sites become occupied after a certain amount of MB dye has occupied them, making it impossible for new dyes to adsorb. In studies on the removal of azo dyes, Yılmaz et al.^72^ and Khatibi et al.^73^ displayed comparable outcomes.

#### Effect of initial MB dye concentration

**Figure 8:**
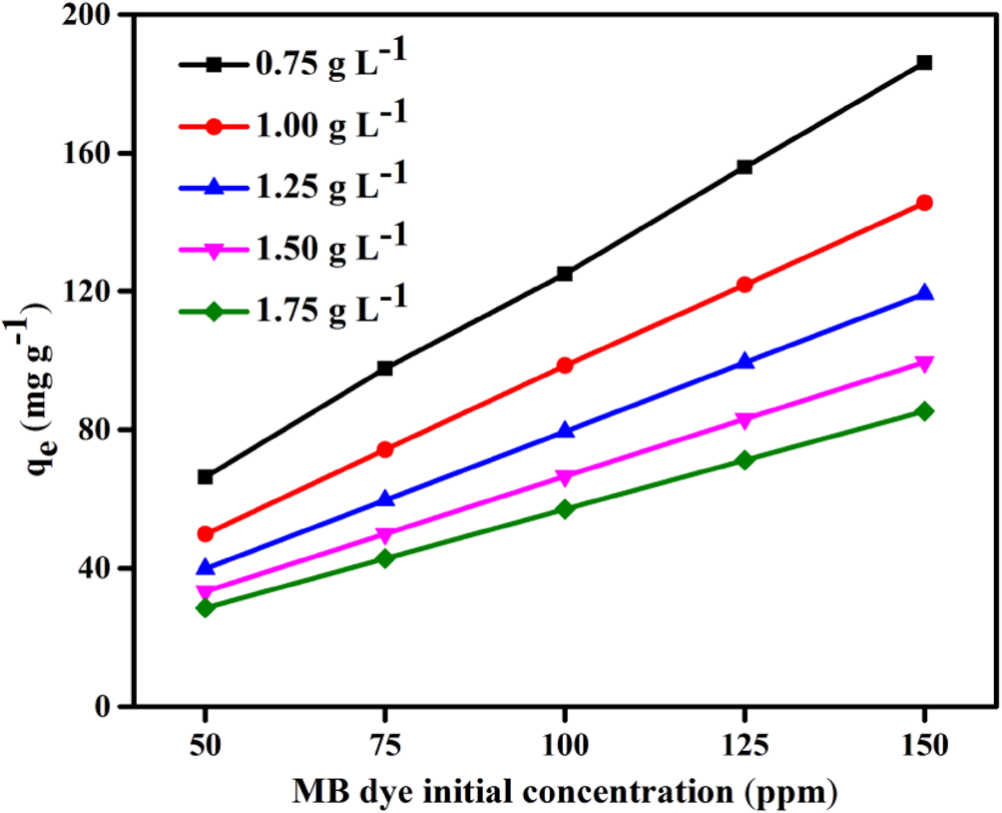
The impact of MB dye initial concentration (50–150 ppm) using DPKB-S doses (0.75–1.75 g L^−1^) on *q*_*e*_ (mg g^−1^) (Temp. = 25 °C).

The MB dye initial concentration is crucial to the adsorption process because it can be used to forecast changes in the equilibrium adsorption capacity (*q*_*e*_). To determine the effect of DPKB-S dose on the steady-state adsorption capacity, the initial MB dye concentration (50, 75, 100, 125, and 150 ppm) and the DPKB-S adsorbent concentration (0.75, 1.00, 1.25, 1.50, and 1.75 g L^−1^) were examined at 25 °C and pH 8 (*q*_*e*_). Figure 8 shows that the amount of MB dye adsorbed at equilibrium (*q*_*e*_) increases at the same initial concentration of MB dye as DPKB-S doses are decreased. As illustrated in Fig. 8, the adsorption capacities of MB dye at equilibrium (*q*_*e*_) were calculated by using DPKB-S adsorbents at different doses (0.75–1.75 g L^−1^). For initial MB dye concentrations (50, 75, 100, 125, and 150 ppm), these values range from 66 to 186, 50 to 145, 40 to 119, 33 to 100, and 29 to 85 mg g^−1^, respectively. Figure 7 illustrates how DPKB-S's MB dye adsorption capacity (*q*_*e*_) increases to equilibrium in solutions with higher initial MB dye concentrations. It was observed that adsorption capacity (*q*_*e*_) values decreased depending on the increase in DPKB-S adsorbent dose. Thus, it is evident that the initial concentration of the MB dye was crucial for its adsorption from its water solution. Shoaib et al.^74^ observed a similar pattern in their study on the adsorption of the dye Direct Blue 86. When MB dye molecules adhere to the DPKB-S adsorbent, they are first exposed to the boundary layer effect. Because the adsorbent is porous, they progressively converge as they diffuse from the boundary layer film to the DPKB-S surface.

#### Effect of DPKB-S adsorbent dosage on MB dye adsorption

**Figure 9:**
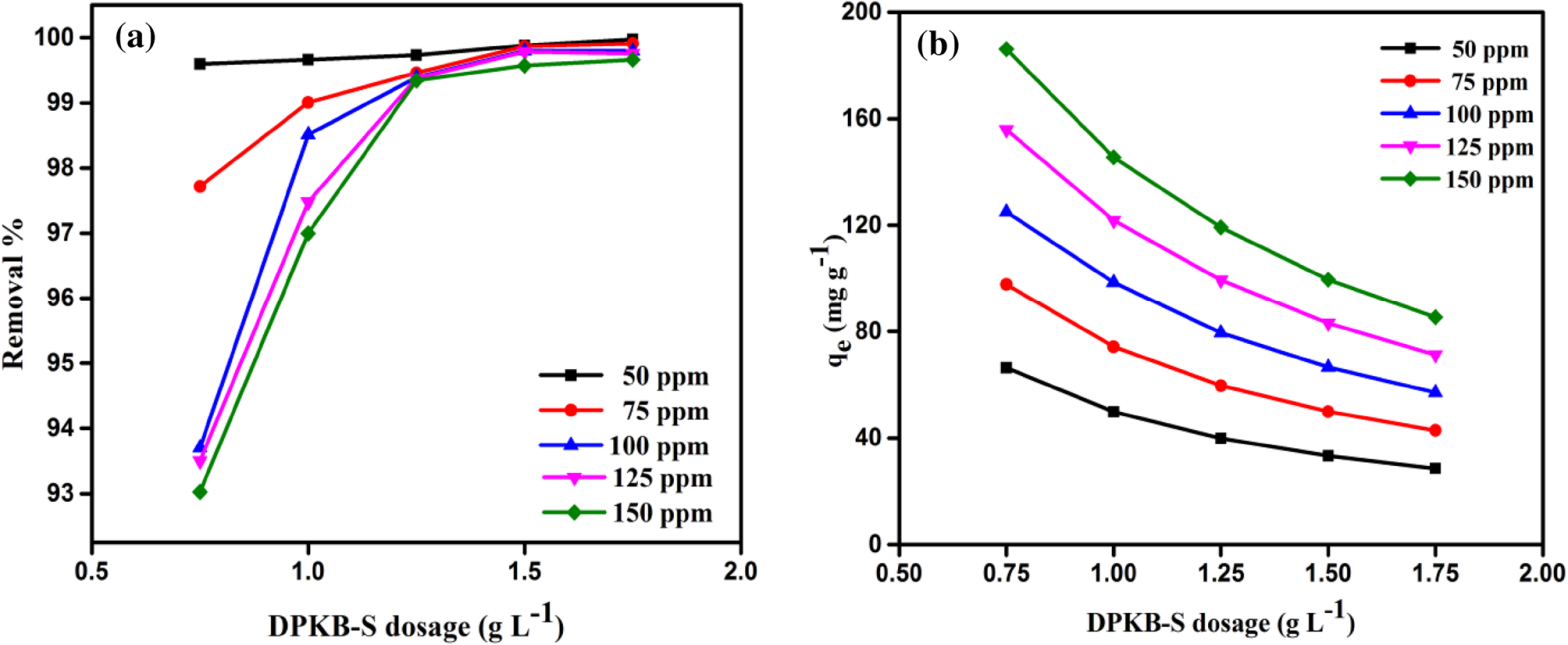
The impact of DPKB-S various doses (0.75–1.75 g L^−1^) of various initial MB dye concentrations (50–150 ppm) (**a**) on removal %; (**b**) on *q*_*e*_ (mg g^−1^), (Temp. = 25 °C).

By adjusting the DPKB-S dosage from 0.75 to 1.75 g L^−1^, utilizing MB dye initial concentration of (50–150 ppm), solution temperature (25 °C), contact time (150 min) at an initial pH of 8.0, the DPKB-S adsorbent dosage effect on removal of MB dye was examined. The results are illustrated in Fig. 9a,b. Experimental results show that when DPKB-S adsorbent dosage is increased, the amount of MB dye adsorbed at equilibrium (*q*_*e*_) decreases (Fig. 9b), but the MB dye elimination percentage slightly increases (between 93–100%) (Fig. 9a). When highly concentrated MB dye molecules are present, the active sites on the DPKB-S surface fill up quickly, causing the release. Thus, 93–99% of the MB dye was removed, when the amount of DPKB-S adsorbent was increased from 0.75 to 1.75 g L^−1^ for initial MB dye concentrations of 50, 75, 100, 125, and 150 ppm, respectively, the amount of MB dye adsorbed at equilibrium (*q*_*e*_) decreases from 99.97 to 99.59, 99.90 to 97.72, 99.79 to 93.70, 99.76 to 93.50, and 99.66 to 93.02 mg g^−1^. It was discovered that 1.75 g L^−1^ DPKB-S dosage showed the lowest adsorption quantity at equilibrium (*q*_*e*_).

#### Temperature impact on MB dye removal %

**Figure 10:**
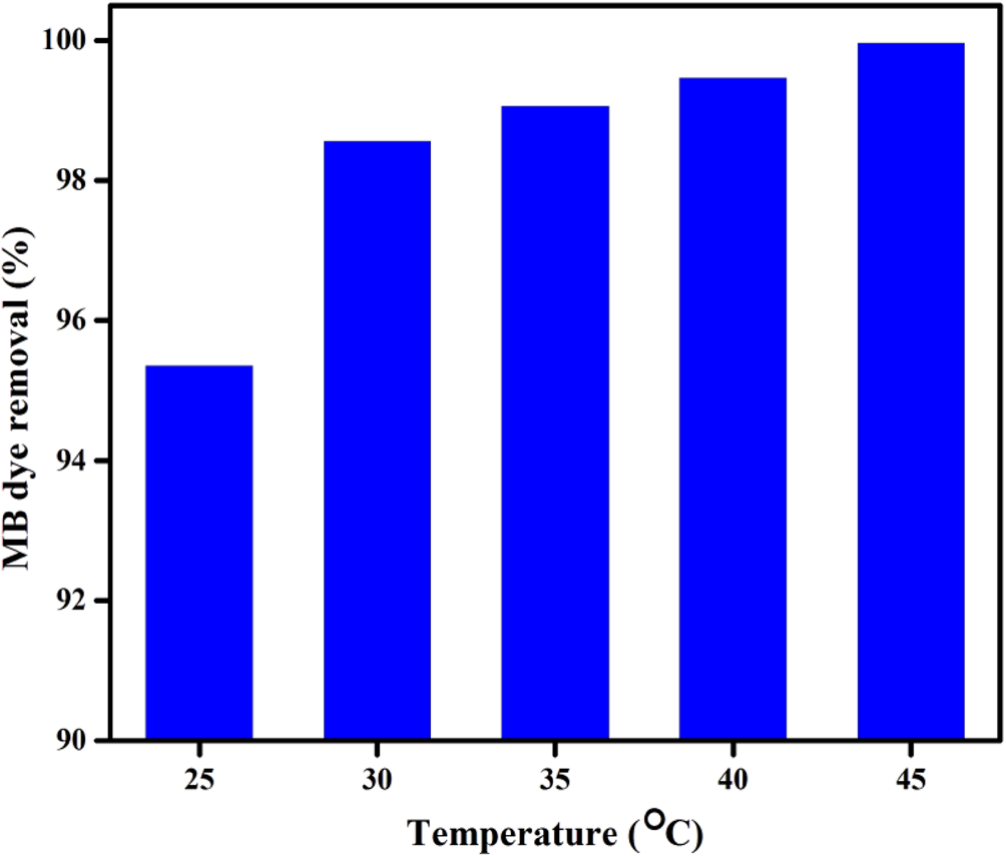
Temperature effect on the adsorption capacity of DPKB-S (0.75 g L^−1^) at pH 8, MB dye (50 ppm) after 150 min.

Figure 10 displays the experimental results of a study that looked at the impact of temperature on the removal of MB dye onto the DPKB-S surface between 25 and 45 °C. The figure demonstrates that as temperature increased, there was a slight increase in the percentage of dye adsorbed onto the DPKB-S surface. This could be the result of MB dye molecules' slight increase in, driving force or kinetic energy, as the adsorption temperature rose from 25 to 45 °C. Increasing the temperature facilitated the diffusion, interaction and adsorption of more MB dye molecules to the active sites on the DPKB-S surface.

### Adsorption isotherms

**Figure 11:**
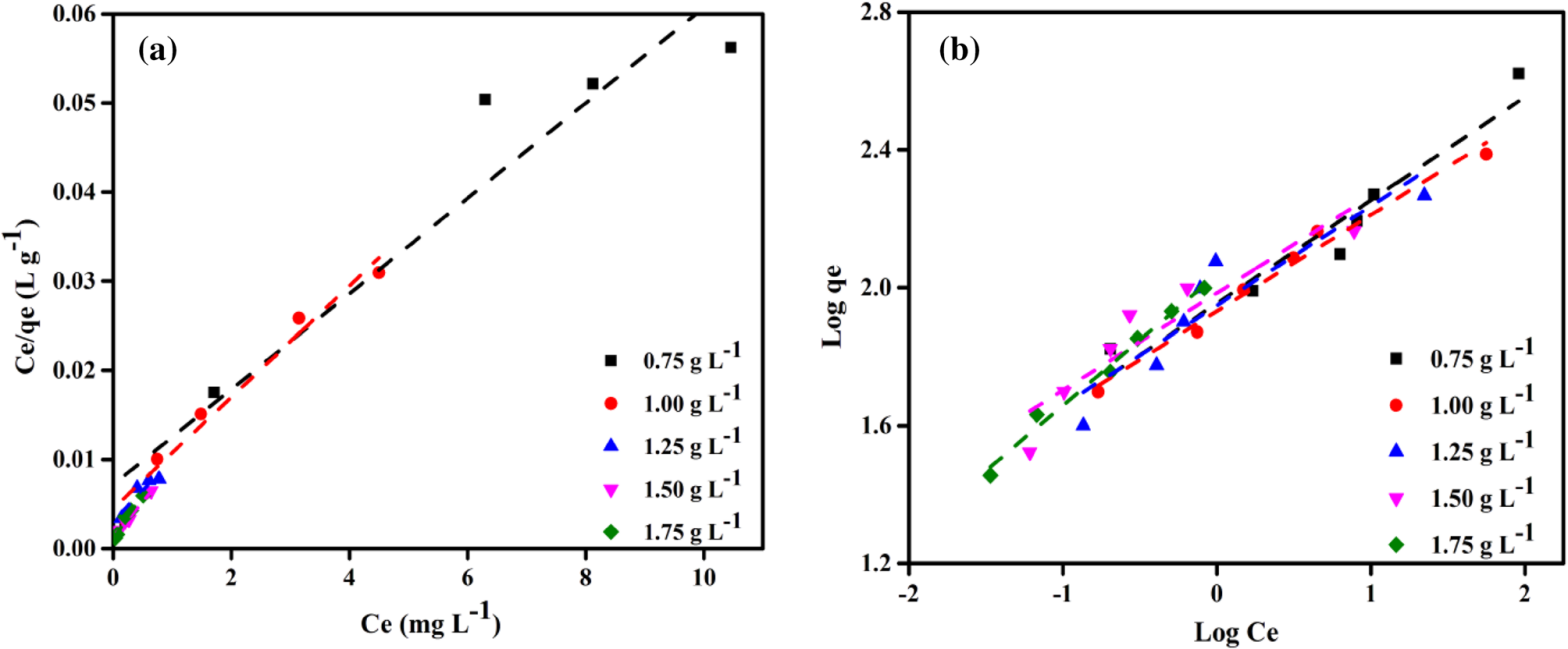
(**a**) LIM, (**b**) FIM profiles for MB dyes of initial concentration (50–150 ppm) on DPKB-S doses (0.75–1.75 g L^−1^) at (25 °C).

By connecting the adsorption isotherm to the equilibrium time, *C*_*o*_ (mg L^−1^) and* q*_*e*_ (mg g^−1^), and to these parameters, it is possible to understand how the adsorbate molecules partition between the liquid and solid phases^75^. The ideal quantity of adsorbent to utilise is determined by using isotherm data and the molecular fraction of the adsorbate distributed in equilibrium (*q*_e_) between solid–liquid phases. The interaction between DPKB-S and MB dye was examined in this study using the Langmuir (LIM) and Freundlich (FIM) isotherm models. The adsorption isotherm describes the diffusion of the adsorbate (MB dye) with the adsorbent (DPKB-S). The LIM is the term for nonlinear monolayer sorption onto homogeneous surfaces without interfering with the adsorbed particles. The link between the solute adsorbed on the adsorbent surfaces and the starting concentration of MB dye is explained by the equilibrium condition in this model. Nonetheless, the heterogeneity of the nonlinear sorption of adsorbents is explained by the Freundlich isotherm model, which is applicable for nonlinear monolayer sorption on heterogeneous surfaces and interacting with adsorbed molecules. The reciprocal interactions between MB dye and DPKB-S were explained by isotherm analyses, the results of which are shown in Fig. 11. MB dye capture through DPKB-S was observed to increase steadily as the initial pollutant concentrations increased. On the other hand, the competition between MB dye and the limited activity sites grew as the number of pollutants increased, which resulted in a slow increase in MB dye removal until equilibrium. The correlation coefficient values for the Langmuir isotherm for the DPKB-S adsorbent concentrations under investigation are displayed in Table 3 as R^2^ (0.810–0.995), *Q*_*m*_ (99.01–185.19), and *K*_*L*_ (0.8–9.2). The date palm kernel biochar-sulfur (DPKB-S) adsorption capacity was determined using the LIM, which assumes that adsorption occurs at homogenous adsorption sites on the surface and that there are no interactions between the molecules of the pollutants that have been adsorbed^76^. Table 3 displays the values of *K*_*F*_ (51.80–135.40), and the correlation coefficient *R*^2^ (0.929–0.992) for the Freundlich isotherm (FIM). In the FIM, it is demonstrated that MB dye exhibits heterogeneous adsorption on the surface of DPKB-S. It is generally accepted that the FIM is the model that best covers MB dye adsorption in DPKB-S at low concentrations (0.75–1.25 g L^−1^) and the Langmuir model at high concentrations (1.5–1.75 g L^−1^). Furthermore, the highest amount of MB dye that DPKB-S could remove was 1512.3 mg g^−1^.

**Table 3:** LIM and FIM studies data of adsorption of MB dye onto DPKB-S adsorbent (MB dye = (50–150 ppm), adsorbent doses = (0.75–1.75 g L^−1^), Temp. = (25 °C)).

Isotherm model	Parameters	DPKB-S adsorbent doses (g L^−1^)				
		0.75	1.00	1.25	1.50	1.75
Langmuir	*Q*_*m*_ (mg g^−1^)	185.19	161.29	144.93	125.00	99.01
	*K*_*L*_ × 10^3^	0.8	1.4	2.2	6.2	9.2
	*R*^2^	0.926	0.975	0.810	0.995	0.977
Freundlich	*Q*_*m*_ (mg/g)	145.2	381.4	1512.3	1380.4	820.0
	*K*_*F*_ (mg^1-1/n^ L^1/n^ g^−1^)	51.8	86.0	110.3	135.4	111.2
	*R*^2^	0.929	0.992	0.957	0.946	0.972

### Investigate the best-fit isotherm model

To select the best model to fit the adsorption of MB dye on DPKB-S, correlation coefficients (*R*^2^) for the LIM and FIM were compared. Another method to determine the most suitable isotherm models is to compare various error function values. To figure out the error distribution between the estimated isotherm models and the equilibrium values, the following error functions were primarily used: root mean square errors (RMS), Chi-square error (X^2^), the sum of the errors squared (ERRSQ), average percent errors (APE), the average relative error (ARE), the sum of absolute errors (EABS), Marquardt's percent standard deviation (MPSD), and hybrid error function (HYBRID)^77^. The error function results showed that the Linear-Langmuir isotherm model is the most appropriate (Table 4).

**Table 4:** The isotherm models' error function values correspond most closely to the experimental equilibrium data on the MB dye adsorption on DPKB-S.

Isotherm model	APE (%)	X^2^	Hybrid	ERRSQ	MPSD	ARE	EABS	RMS
Linear-Langmuir	0.101	1.355	5.892	115.783	0.525	0.101	53.801	0.504
Freundlich	0.553	39.234	170.581	3218.233	2.884	0.553	283.647	2.766

### Adsorption kinetic studies

The mechanism of MB dye adsorption onto DPKB-S is explained using kinetic models. The Lagergren model, or pseudo-first-order (PFO) model, is a kinetic model that illustrates the sorption kinetics as a function of contact time. The pseudo-second-order (PSO) kinetic model suggests a close relationship between the chemical reaction rate and the active sites on the surface of the biochar. However, the internal adsorption of the adsorbent in an aqueous solution is interpreted by the intraparticle diffusion (IPD) kinetic model.

**Figure 12:**
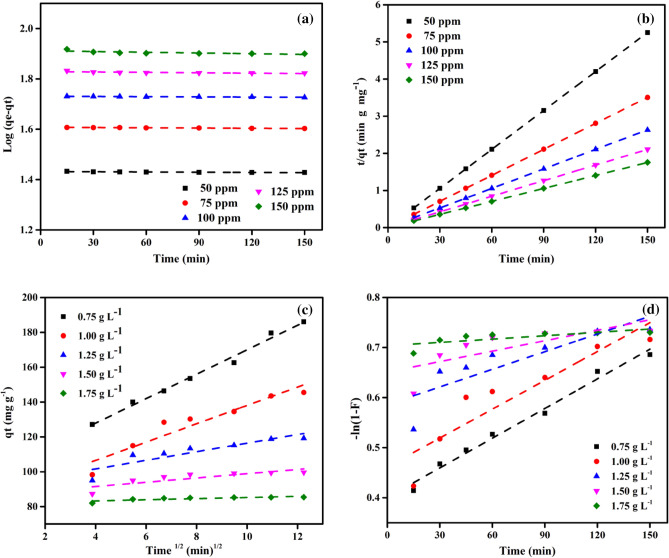
(**a**) PFO, (**b**) PSO, (**c**) intraparticle diffusion, and (**d**) film diffusion kinetic models of adsorption of MB dye by DPKB-S adsorbent (*C*_0_ = (50–175 ppm), Adsorbent dose = (1.75 g L^−1^), Temp. = 25 °C).

The absorption of DPKB-S for MB dye was investigated through kinetic studies (Fig. 12). DPKB-S removed a significant amount of MB dye in the first 15 min (Fig. 7), as can be seen. This is probably because DPKB-S offered a lot of adsorption sites. The equilibrium of MB dye binding to DPKB-S was reached in 150 min. as the adsorption sites were filled. PFO, PSO, IPD, and FD models were utilized to simulate the uptake effects to quantitatively investigate the binding process of MB dye on DPKB-S^78–80^.

Kinetic model applicability was assessed using the coefficient (*R*^2^); a higher *R*^2^ value suggested better applicability. Adsorption kinetics gives an idea of the biochar's time-dependent adsorption capacity. Calculating the rate constant (*k*_1_) and equilibrium adsorption capacity (*q*_*e*_) from the linear graph of Log(*q*_*e*_ − *q*_*t*_) values against time (*t*) is shown in Fig. 12a. The values of *k*_1_ (0.02–2.07), *q*_*e*_ (27.08–252.70), and *R*^2^ (0.504–0.983) for the PFO kinetic were ascertained by examining the linear form of the PFO kinetic (Table 5).

**Table 5:** PFO and PSO kinetic model results of MB dye adsorption by DPKB-S (Initial concentration = (50–150 ppm), adsorbent doses = (0.75–1.75 g L^−1^), Temp. = (25 °C).

Parameter			PFO			PSO			
DPKB-S (g L^−1^)	MB dye (ppm)	*q*_*e*_ (exp.)	*q*_*e*_ (calc.)	*k*_*1*_ × 10^3^	R^2^	*q*_*e*_ (calc.)	*k*_*2*_ × 10^3^	*h*	R^2^
0.75	50	66.40	62.49	0.46	0.664	66.67	19.91	88,495	1.000
	75	97.72	106.15	1.15	0.805	100.00	2.26	22,624	1.000
	100	124.94	158.45	1.61	0.869	129.87	0.88	14,836	0.998
	125	155.83	192.18	1.61	0.741	161.29	0.95	24,752	0.999
	150	186.05	252.70	2.07	0.983	200.00	0.36	14,388	0.993
1.00	50	49.83	46.81	0.09	0.870	50.00	6.25	15,625	1.000
	75	74.26	74.40	0.69	0.504	75.19	8.71	49,261	1.000
	100	98.51	102.83	0.69	0.886	100.00	3.12	31,152	1.000
	125	121.85	131.86	1.15	0.667	125.00	2.11	33,003	1.000
	150	145.50	179.35	1.84	0.847	153.85	0.66	15,527	0.999
1.25	50	39.89	37.25	0.07	0.853	40.00	96.15	153,846	1.000
	75	59.68	56.35	0.12	0.608	59.88	4.50	16,129	1.000
	100	79.51	78.04	0.69	0.659	80.00	11.41	72,992	1.000
	125	99.38	100.79	0.69	0.681	101.01	4.05	41,322	1.000
	150	119.21	129.15	1.38	0.739	121.95	1.84	27,322	1.000
1.50	50	33.29	31.31	0.07	0.880	33.33	121.62	135,135	1.000
	75	49.93	46.92	0.09	0.906	50.00	74.07	185,185	1.000
	100	66.53	62.57	0.12	0.572	66.67	43.27	192,307	1.000
	125	83.15	80.70	0.46	0.518	84.03	13.11	92,592	1.000
	150	99.57	99.88	0.69	0.586	101.01	5.03	51,282	1.000
1.75	50	28.55	27.08	0.09	0.658	28.57	153.13	125,000	1.000
	75	42.82	40.51	0.07	0.729	42.92	68.72	126,582	1.000
	100	57.03	53.85	0.07	0.805	57.14	69.60	227,272	1.000
	125	71.25	67.55	0.14	0.675	71.43	38.43	196,078	1.000
	150	85.42	81.58	0.02	0.723	85.47	20.43	149,253	1.000

The PSO kinetic is based on the dynamics of mass transfer. Plotting *t/q*_*e*_ vs *t* yields the amount of MB dye adsorbed at equilibrium (*q*_*e*_) and the PSO kinetic constant, *k*_*2*_. This is demonstrated in Fig. 12b. The linear form of PSO kinetics was represented by the values of *k*_*2*_ (0.36–153.13), *q*_*e*_ (28.57–200.00), and R^2^ (0.993–1.000), as shown in Table 5.

The fact that the lines in the qt and root time (*t*) graph in Fig. 12c pass through the origin means that, according to Weber and Morris theory, adsorption occurs with the intra-particle diffusion step^81^. On the other hand, it is believed that film diffusion (FD) controls the rate of the adsorption process when the drawn lines do not pass through the origin (that is when the *C* value is high). Figure 12c shows the Webber-Morris adsorption line for the adsorption of MB dye on DPKB-S adsorbent at different adsorbent dosages at varying initial MB dye concentrations. As demonstrated in Table 6, IPD kinetic analysis provided *K*_*dif*_, *C*, and *R*^2^ values for the adsorption of MB dye onto DPKB-S as 0.033–7.020, 28.184–108.230, and 0.603–0.990, respectively. The PSO kinetic model (*R*^2^ = 1.000) out of the four kinetic models best fits the dynamics of MB dye sorption onto DPKB-S. The findings of this study are similar to those of other investigations that used wastewater sludge for MB dye adsorption^78^.

**Table 6:** IPD and FD kinetic model results of MB dye adsorption by DPKB-S (*C*_0_ = (50–150 ppm), DPKB-S doses = (0.75–1.75 g L^−1^), Temp. = (25 °C).

DPKB-S (g L^−1^)	Parameter		IPD			FM	
	MB dye (ppm)	*q*_*e*_ (exp.)	*K*_*dif*_	*C*	R^2^	*K*_*FD*_	R^2^
0.75	50	66.40	0.306	63.104	0.748	0.000	0.628
	75	97.72	1.977	75.721	0.892	0.001	0.804
	100	124.94	3.927	78.819	0.919	0.002	0.869
	125	155.83	4.063	108.230	0.862	0.001	0.794
	150	186.05	7.020	99.813	0.990	0.400	0.985
1.00	50	49.83	0.076	48.969	0.947	0.000	0.869
	75	74.26	0.795	65.933	0.629	0.001	0.503
	100	98.51	1.344	83.095	0.951	0.001	0.886
	125	121.85	2.360	95.996	0.767	0.001	0.667
	150	145.50	5.264	85.437	0.907	0.002	0.847
1.25	50	39.89	0.050	39.326	0.932	0.000	0.853
	75	59.68	0.127	58.252	0.720	0.000	0.608
	100	79.51	0.576	73.458	0.724	0.000	0.594
	125	99.38	1.338	84.881	0.793	0.001	0.681
	150	119.21	2.468	91.775	0.809	0.001	0.729
1.50	50	33.29	0.037	32.869	0.944	0.000	0.888
	75	49.93	0.629	49.212	0.952	0.000	0.906
	100	66.53	0.024	21.912	0.943	0.001	0.518
	125	83.15	0.562	77.295	0.603	0.000	0.554
	150	99.57	1.227	86.619	0.707	0.001	0.639
1.75	50	28.55	0.033	28.184	0.902	0.000	0.810
	75	42.82	0.049	42.186	0.934	0.000	0.973
	100	57.03	0.054	56.333	0.979	0.000	0.969
	125	71.25	0.153	69.553	0.763	0.000	0.644
	150	85.42	0.329	81.928	0.683	0.000	0.555

A PSO model with high *R*^2^ values was used to show how well MB dye eliminated DPKB-S, and the data came from Tables 5, 6. The PSO model states that the adsorption is controlled by the quantity of MB dye on DPKB-S's surface and that a chemical adsorption process may be the rate-limiting step^78,82^.

### Adsorption mechanism of MB dye by DPKB-S

**Figure 13:**
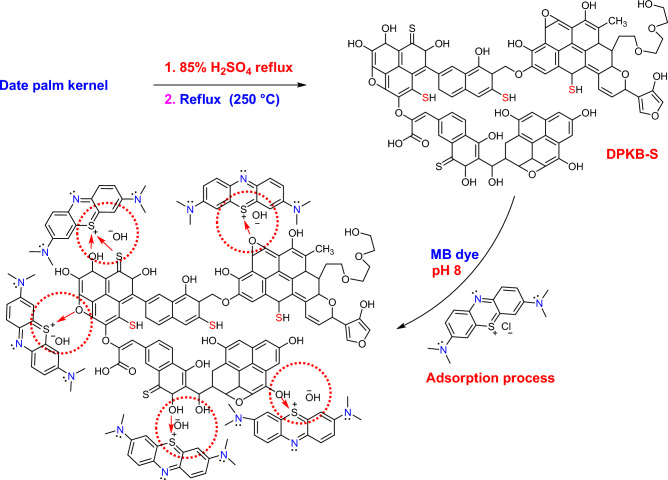
The likely mechanism by which the MB dye is adsorbed onto the DPKB-S biochar.

Figure 13 explains the likely mechanism via which DPKB-S absorbed the MB dye ions. following the 85% H_2_SO_4_ dehydration of the date palm kernel raw material. According to FTIR analysis, a variety of functional groups, including C=O, COOH, C–O–C, hydroxyl O–H, C–S, and SH groups, developed on the surface of the adsorbent (DPKB-S). Because of the electrostatic interaction between the nitrogen lone pair on the DPKB-S surface and the positive charge on the sulphur atom of the MB dye, the adsorption mechanism of the MB dye ions in a base medium (pH 8) can be accomplished through physical interaction. Once the surface charge became positive, the basic pH of the base medium attracted hydroxyl ions.

In an alkaline environment, the surface of biochar picks up a negative charge, which attracts positively charged dye molecules^83,84^. Additionally, the OH^−^ ions in the solution interact with the carboxylic and phenolic functional groups on the surface of the biochar to form negatively charged sites that can further adsorb positively charged dye molecules. Additionally, dye molecules are more soluble at a basic pH, which facilitates their diffusion through the pores in the biochar and their attachment to the adsorption sites. Biochar is an excellent method for removing colour from industrial effluent because the basic pH is essential for encouraging the adsorption of dye molecules onto the material. The adsorption of ionizable organic molecules to the positively charged surface of the biochar via electrostatic interaction is the most significant process^84^. How successfully an aqueous solution attracts or repels impurities depends on its pH and ionic strength^20,84^.

Furthermore, the capacity of organic contaminants in industrial effluent to adsorb is influenced by the pH of the solution^72^. Parshetti et al.’s study^85^ examined the use of food waste-derived biochar in the adsorption of textile colours in wastewater. They found that an alkaline pH enhanced the adsorption of dyes. The significant interaction between the negatively charged sites on the biochar surface and the positively charged dyes explained it^85^. However, since there was an excess of H^+^ at pH 3, which competed with the positive charges of the dye, it was less successful at adsorbing organic dye^85^. Tsai and Chen^86^ and Xu et al.^87^ have noted that pH has an impact on biochar's capacity to absorb materials. As a result, the charged sites are altered by the pH of the solution, which alters the ability of organic and inorganic contaminants from industrial effluent to adsorb on biochar.

### Comparing the outcomes with those documented in the literature

The efficacy of removing MB dye using various adsorbents was compared with the Date palm kernel Biochar-Sulfur (DPKB-S) adsorbent in the literature review that is summarized in Table 7. The results indicated that DPKB-S is super efficient in removing MB dye compared to the published results in the literature.

**Table 7:** Comparison of *Q*_m_ and removal % of MB dye of different adsorbents.

Adsorbent name	*Q*_m_ (mg g^−1^)	Removal (%)	Ref
Oil palm fibres	382.32	–	^33^
Date palm seeds	612.10	–	^35^
African almond leaves biochar (PALB)	263.95	97.00	^70^
Activated carbon of *Coriandrum sativum*	94.97	97.78	^78^
Co-pyrolysis of sewage sludge (SS) and lignin	154.06	–	^88^
AC from rubber seeds	769.20	99.00	^89^
AC from cardboard tube waste	182.48	99.70	^90^
AC from *Calicotome* *villosa* wood	169.80	97.00	^91^
Coconut shell	156.25	–	^92^
Activated carbon from eucalyptus	977.00	99.60	^93^
Microalgal biomass	113.00	89.78	^94^
DPKB-S	1512.30	100.00	[This work]

### Regeneration of DPKB-S

**Figure 14:**
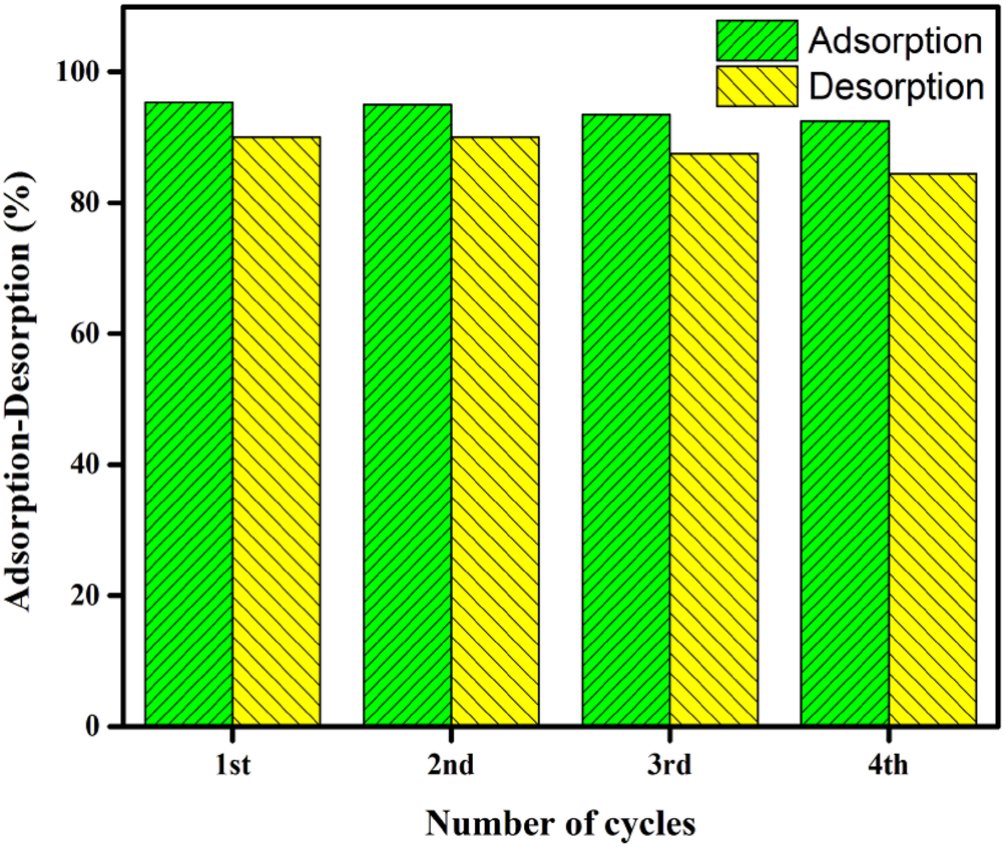
The percentage of MB dye that was desorption from DPKB-S using 0.1 M NaOH and 0.1 N HCl, as well as the MB dye adsorption cycles utilising regenerated DPKB-S.

To evaluate the feasibility and reusability of DPKB-S as an adsorbent, desorption experiments of the MB dye from the loaded DPKB-S were studied using 0.1N HCl and 0.1 M NaOH as the elution medium. In this instance, increasing the regeneration cycles resulted in a decrease in the desorption % (Fig. 14). The regenerated DPKB-S was used to carry out four successive adsorption/desorption cycles. The adsoption-desorption cycle of the obtained DPKB-S adsorbent decreased by only 2.98% from its initial efficiency after four cycles. According to Fig. 14, it implies that it might be applied as a long-lasting method for removing MB dye.

## Conclusion

Date Palm Kernel Biochar-Sulfur (DPKB-S) was produced to study the efficacy of methylene blue adsorption from water. It investigated the characterization of DPKB-S and the impacts of pH, initial MB dye concentration, DPKB-S biochar doses, contact time, and temperature on the adsorption. DPKB-S was obtained from date palm kernel by dehydration of biochar with 85% sulfuric acid. This aids in the mass diffusion of MB dye and creates a surface that can be used for the MB dye removal process. The equilibrium sorption for the MB dye gained an adsorption capacity of 1512.30 mg g^−1^ using DPKB-S at pH 8 for 100 ppm of MB dye concentration and at 25 °C. Freundlich model is the model that best covers MB dye adsorption in DPKB-S at low concentrations (0.75–1.25 g L^−1^) and the Langmuir model at high concentrations (1.5–1.75 g L^−1^). Pseudo-second-order has been preferred as the best-fitted kinetic model. The produced DPKB-S biochars compromised only 2.98% in effectiveness even after four uses. The work indicated that DPKB-S is a promising adsorbent candidate for the removal of MB dye from wastewater.

## Data Availability

The datasets used in this investigation are accessible for review upon request from the paper's corresponding author.
